# Precepting Through Perinatal Emergencies: A Simulation‐Based Training for Midwifery Educators

**DOI:** 10.1111/jmwh.70024

**Published:** 2025-09-13

**Authors:** Susanna R. Cohen, Kimberly Calkins, Jennifer E. Kaiser, Heidi Breeze Harris, Elizabeth Auricchio, Julie Blumenfeld

**Affiliations:** ^1^ OBGYN Department LIFT Simulation Lab, ASCENT Center, University of Utah Salt Lake City Utah; ^2^ PRONTO International Seattle Washington; ^3^ ASCENT Center, University of Utah Salt Lake City Utah; ^4^ Nurse‐Midwifery Program, Division of Advanced Nursing Practice School of Nursing, Rutgers, The State University of New Jersey Newark New Jersey

**Keywords:** education (professional), midwifery, mentoring, simulation training

## Abstract

The growth of the midwifery model of care depends on the preparation of new midwives, which necessitates skilled midwifery clinical preceptors. The University of Utah, Rutgers University, and PRONTO International supported by the New Jersey Department of Health, created the *Precepting Through Perinatal Emergencies Workshop*. We developed this sustainable in‐person and virtual preceptor educational content through iterative feedback and pilot testing with active New Jersey midwifery preceptors. The preceptor training centered around introducing preceptors to evidence‐based educational tools like the Educational Time Out, a teaching strategy using guided discovery learning concepts, goal setting, peer coaching strategies, and adult learning theories to enhance communication and debriefing skills. The in‐person, highly interactive workshops included didactic lessons, role‐plays, and 2 high‐fidelity person‐centered simulation scenarios and debriefs using the model developed by PRONTO International. The initial workshop's success led us to create a facilitation workshop for preceptors to learn how to train others and 3 online asynchronous modules to augment the learning. Midwifery preceptors who completed the facilitator training were equipped with the requisite skills, knowledge, and supplies needed to repeat the training in their home facilities.

## BACKGROUND

Midwifery‐led care, which centers patients and focuses on physiologic birth, is associated with greater satisfaction and improved perinatal outcomes compared with other US care models.^1^ Growing the midwifery workforce is a strategic approach to reducing disparities in maternal and infant health.^2^ However, this growth is limited due to a lack of access to qualified midwifery preceptors, who are central to the clinical training of midwifery students.^3^ Midwifery preceptors play an essential role in the training and education of midwifery students, assisting them to safely actualize the content learned in the classroom and simulation lab.^4^ For midwives to develop proficiency in the role of preceptor, which is distinct from their role as clinicians, they need education and support.^4^ To effectively work with students, preceptors need to develop a foundational skillset that includes strategies to support adult learners, address challenges in the clinical setting, and effectively convey feedback.^5^ Usual challenges associated with teaching and learning in the clinical environment may be exacerbated when the preceptor and learner face stressful situations.^5^ Despite research demonstrating that trained preceptors contribute to developing students’ capacity and self‐confidence^6^ and preceptors’ affirmation that educational support facilitates effective precepting,^7^ there is a paucity of opportunities for hands‐on education and training on precepting. Most preceptor education comes in written content with occasional in‐person orientation, which is necessary but insufficient to support midwifery preceptor learners in acquiring and practicing the required skills to improve confidence and competence in precepting students.

  

Continuing education (CE) is available for this article. To obtain CE online, please visit http://www.jmwhce.org. A CE form that includes the test questions is available in the print edition of this issue.

## INTRODUCTION

In New Jersey, a statewide strategic plan to improve perinatal health outcomes includes expansion of the midwifery workforce as one of its strategies.^8^ This has been actualized by establishing the New Jersey Midwifery Education Project at Rutgers University School of Nursing, funded by the New Jersey Department of Health. The Project's objectives include expanding access to midwifery education, increasing and improving training for students in the clinical setting, and growing and diversifying the midwifery workforce.

**QUICK POINTS**

✦ There is a need for evidence‐based training and education specific to midwifery precepting.
✦ Highly realistic simulation can support preceptors in the practice and integration of learning strategies.
✦ The Educational Time Out can help students before, during, and after a clinical experience.
✦ The Coaching Conversation Guide can support midwifery preceptors and students in improving communication and enhancing clinical learning experiences.

One of the Project's initiatives was to develop and fund midwifery preceptor‐specific education and training. Throughout the spring and summer of 2023, the Project hosted the Midwifery Preceptor Workshop Series, sharing updates on best practices in teaching midwifery in clinical settings. It addressed topics such as suturing and perineal repair, consent and trauma‐informed care, integrating equity, inclusion, and cultural humility into precepting, and precepting through perinatal emergencies. The *Precepting Through Perinatal Emergencies Workshop* started an ongoing partnership between the Project and the University of Utah. Their interprofessional collaborative, comprising 3 midwives, an obstetrician‐gynecologist, and a simulation curriculum specialist, created an innovative training for midwifery preceptors. The initiative's overarching goal was to enhance preceptors’ skills and confidence as educators in the intrapartum setting, especially during birth emergencies. The program integrates the learnings from various evidence‐based practices, including (1) the Educational Time Out (ETO), a teaching strategy that uses concepts of guided discovery learning and goal setting^9^, ^10^; (2) high‐fidelity person‐centered simulation (using the PRONTO method); and (3) peer coaching strategies and adult learning theories to enhance communication and debriefing skills.^11^, ^12^, ^13^, ^14^ In this article, we review the program goals, objectives, and content and highlight the role of simulation and debriefing in supporting preceptors in integrating evidence‐based learning practices into their coaching and teaching of student midwives.

## PROGRAM GOALS AND OBJECTIVES

*Precepting Through Perinatal Emergencies: A Training for Midwives* was a single‐day, 8‐hour workshop for midwifery preceptors. The training goals were to increase midwifery preceptors’ capacity to be effective, empathetic, and motivational; establish and maintain a positive learning environment for student midwives; and guide learners during high‐stress events. The program allowed midwifery preceptors to practice mentoring, peer coaching, student goal setting, and debriefing in a simulated environment. Learning strategies and curriculum content were chosen to match the needs of adult learners. The program included didactic teaching, role‐play, high‐fidelity simulation and debriefing, and group discussion to create a learning environment that engaged learners and created a learning space that encouraged conversation, peer‐to‐peer learning, and productive struggle.^15^ Each curricular component scaffolds learning—beginning with basic theory, moving to low‐stakes pair role‐play, and culminating in 2 high‐fidelity simulations integrating didactic content with highly realistic practice.

## CURRICULAR CONTENT AND THEORY

Curricular content was chosen based on a review of evidence‐based practice in student clinical teaching, coaching, and debriefing. The project leaders reviewed the literature for medical, surgical, and midwifery clinical precepting and created a curriculum that reflected the reality of midwifery practice.

### The ETO and Goal Setting

**Figure 1:**
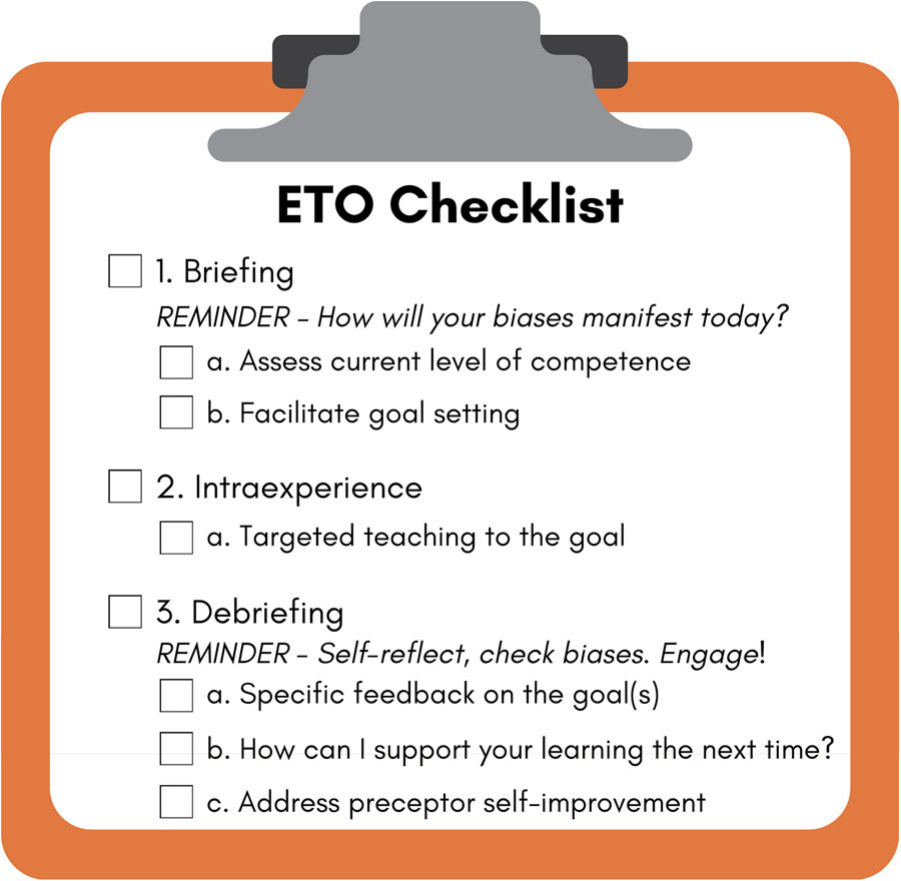
Educational Time Out Checklist Abbreviation: ETO, Educational Time Out.

The program design team used the ETO as the didactic backbone and educational best practice of our training. The ETO provides preceptors with a structured approach and tool to inform their precepting in clinical scenarios and can help improve in‐the‐moment teaching skills and student learning.^9^, ^10^, ^16^ See Figure 1 for midwifery education. Initially developed in surgical education, this tool uses the briefing, intraoperative teaching, debriefing (BID) framework to guide learners through a surgical experience.^10^ The BID framework is predicated on the guided discovery learning (GDL) theory, which posits that expert facilitation through a learning experience results in more rapid acquisition and retention of skills. GDL involves an expert preparing a learner before an experience, offering verbal or manual guidance during the experience, and leading a debrief afterward.^17^ The BID framework in surgical graduate medical education involves a surgeon and resident setting a learning goal before a procedure, the surgeon teaching to the goal during the surgery, and then the surgeon and resident debriefing on the goal following the surgery. Previous studies have found that BID‐based ETO models improved the quality and rates of postoperative feedback, increased levels of resident surgical autonomy, and enhanced educational experiences. A recent study using highly realistic simulation to train gynecologic surgical faculty on ETO use showed significant improvement in preceptor teaching self‐efficacy and confidence in providing constructive feedback.^9^, ^16^, ^18^ The midwifery project adapted the ETO framework and focused on training preceptors on how to guide students to set meaningful goals. The SMART (Specific, Measurable, Achievable, Relevant, Time‐bound) Goal framework^19^ provided structure for the goal‐setting content and role‐plays. For the ETO to be most beneficial, learners needed to set an actionable goal for the upcoming encounter. Without following the SMART framework, learners could choose broad, vague, or unachievable goals such as “I want to work on everything” or “I want to care for all of the patients,” which would not be specific enough to focus learning for either the preceptor or the student midwife.

Using the ETO during midwifery precepting provides structure to the clinical learning experience and supports students to progress into the role of safe beginning practitioners. A critical adaptation made for the midwifery preceptor learners was incorporating bias‐awareness strategies into the ETO. Implicit bias has been shown to affect the type and number of clinical experiences that students are offered in clinical spaces, the learning goals that are set, and the way feedback is given to students.^20^, ^21^ Bringing awareness to internal thoughts and beliefs can help mitigate their effects on precepting.^22^

### Peer Coaching and Coaching Conversations

In addition to the ETO, the curriculum focused on the interpersonal and communication skills that make precepting more effective—peer coaching, coaching conversations and feedback, and educational empathy. Clinical learning is a collaborative effort between the preceptor and the learner. The peer coaching model offers concrete collaborative skills for the midwifery preceptor. Although midwifery students and preceptors are not technically peers, they are nearly peers. They have been professionals first as nurses or in other areas and, once certified, will be peers again. Building a meaningful professional relationship is important for students, preceptors, and future collaboration. Learning in the clinical setting is a collaborative effort, and coaching centers the adult learner and fosters a culture of professionalism and respectful communication. This approach creates a safe space where concrete communication and feedback skills can help students learn new skills or information, share ideas, solve problems, and reflect on current practices.^11^, ^12^, ^13^, ^23^

Peer coaching engages the learner before, during, and after a patient encounter to help them solidify skills and knowledge. A peer coach ensures their learner's competency and instills confidence in their skills and knowledge through the growing relationship. Our curriculum included a Peer Coaching Conversation Guide, informed by published models, designed especially for midwifery students during intrapartum clinical experiences. The guide was introduced during the training and practiced by the participants during the simulations. See Table 1.

**Table 1:** Peer Coaching Conversation Guide

Time Frame	Conversation Examples
**Setting the stage**	
Assess level of experience	“How many births have you participated in?”
	“How many have you had hands‐on experience with…”
	“In your last 2 or 3 births, how much were you doing independently?”
Assess level of confidence	“How do you feel about (skill)…?”
Role clarification	“Let's clarify our roles for today…”
Setting expectations	“Let's be sure our expectations align…”
Facilitate goal setting	“What would you like to work on today?”
	“Let's set some SMART goals for our day together.”
**During the clinical encounter**	Be an active observer. Pay Attention. Prompt transparent thinking. Be a soothing presence.
**Debrief discussion**	
Reactions	“How did that feel?” or “Any initial reactions?”
Description	“Together we set… (Review SMART Goal)…”
Analysis	“What went well?”
	“What could have gone better?”
Ask permission	“Would you like to hear my suggestions?”
Give suggestions	I saw…
	I like…
	I am curious…
	I wonder…
Facilitate action planning	“What are some take‐aways from this discussion?”
	“What is something you would do again, or do differently next time?”
Coach‐improvement	“What can I do again or do differently the next time we work together to support your learning?”

Abbreviation: SMART, Specific, Measurable, Achievable, Relevant, Time‐bound.

Additionally, the curriculum included material on giving feedback,^13^ understanding a preceptor's circle of safety,^24^ especially during an emergent clinical situation, and the bidirectionality of feedback.^25^ Group discussions included reflection on the safety of the patient, the student, and the preceptor and how that boundary shifts due to patient situation, preceptor experience, student competency, and other facility and clinical factors. The design team also included a section on the learning principle of productive struggle and how letting learners struggle^26^ can increase confidence and solidify skills.

In one final curricular innovation, the team adapted clinical empathy skills often used with patients into a set of newly named *educational empathy* skills that midwifery preceptors can practice with their learners. Clinical empathy is defined as understanding what a patient says and feels and effectively communicating that understanding to the patient.^27^ This project encouraged preceptors to use the highlighted skills to understand what their students say and feel and effectively communicate that understanding to the students. These skills encourage emotional attunement with the learner to create a stronger education alliance. See Table 2 for an overview and examples of educational empathy skills used in the training.

**Table 2:** Educational Empathy Skills

Educational Empathy Skill^a^	Definition	Example
Pacing	The rhythm and cadence of an interaction. The preceptor can track and monitor the needs of the learner and adjust clinical teaching, patient encounters, and feedback sessions.	Learner: This is so much information. Everything is about percentages, and I can't keep them all straight. There are so many different tests! (sigh) Preceptor: You're so right. It is a lot of information. I wonder if I'm throwing too much at you at once. I'm going to pump the breaks. Let's see what questions you have.
Reflecting Feeling	The act of determining a person's feelings and reflecting them back to them through validation and offering new language as needed.	Learner: I see that the person we are caring for today has a history of shoulder dystocia in their last birth, and in this pregnancy, they have macrosomia. They really want a vaginal birth, and we have talked about the risks. I have never seen a shoulder dystocia, but I practice the maneuvers in my head before each birth. What do you think we should do? Am I missing something? Preceptor: We can definitely review the management steps of shoulder dystocia today before the birth. You seem pretty worried, and it's not unusual to feel scared at this point in your training. Let's talk a bit about what you're feeling.
Reflecting Meaning	The skill of highlighting the underlying values, attitudes, and beliefs about a topic to raise awareness and identify alternative perspectives as needed.	Learner: I really just want to watch you educate the patient and the family about shoulder dystocia; last time, the preceptor said that I scared the patient, but I was trying to be honest. All the midwives do something different. Provider: It sounds like my support of you as a learner is important, and you are trying to navigate what is the type of patient education that feels authentic to you. Tell me more about what you've been thinking.

^a^ Adapted from clinical empathy.

### Simulation Content

Evidence‐based and compassionate clinical mentoring is essential in educating and training future nurses.^28^ As such, it is essential that all preceptors receive high‐quality professional training and development in clinical educator roles.^28^ The ETO, peer coaching, and circle of safety methods in midwifery precepting can structure the clinical experience. Still, it requires an opportunity for the preceptor to actively practice these skills for optimal retention and replication. Simulation has a well‐established track record in nursing and midwifery education. Yet, few clinical faculty involved in preclinical nursing simulation have had exposure to formal training on educational or learning theories. In previous studies, nursing students have identified the value of support and specific feedback from their educators; however, the same educators felt they were ill‐equipped and lacked sufficient training to provide quality or supportive feedback.^29^ There is an overall paucity of rigorous studies on the effect of simulation or its use for training clinical preceptors. Simulation learning, specifically for preceptor education and training, allows novice preceptors to have an experiential, interactive, and reflective experience in giving and receiving feedback from their fellow preceptor peers.^29^ Simulation training, grounded in evidence‐based learning theory and teaching strategies, can meaningfully impact the learning experience by allowing preceptors and clinical educators to practice delivering formative feedback in clinical settings.^29^

#### PRONTO‐Style Simulation and Team Training

The simulations used in this curriculum were based on those created by PRONTO International. PRONTO designs and conducts simulation‐based training for health care provider teams using an innovative, evidence‐based approach to help learners move from knowledge to practice in managing maternal and newborn emergencies during the intrapartum period.

In addition to strengthening individual provider skills and knowledge, the training reinforces teamwork and communication, interprofessional collaboration, and person‐centered maternal care. PRONTO's unique simulation‐based trainings allow health care provider teams to practice skills in simulated high‐stress environments, ensuring they are prepared to respond efficiently and effectively during an emergency.

For the role of the patient in this scenario, we hired an actor to wear the PartoPants hybrid birth simulator.^30^ Training participants took turns as the midwifery preceptor, the simulated student, and the nursing staff. The goal was for the preceptors to practice coaching the student through goal setting and managing a complicated birth and emergency. The participant who played the student was given guidelines and a script. The goals that the student set were preprogrammed so that the preceptors would have opportunities to practice guiding students with different challenges, including those with both low confidence or overconfidence and those who made clinical errors, all while managing the clinical situation and communicating with the birthing person and their family members. At the beginning of the scenario, the preceptor practiced goal setting with the student, coached them using peer coaching skills during the emergency, and debriefed the student using feedback techniques covered in the didactic sessions at the beginning of the day. See Table 3 for a summary of the 2 simulations used and the roles of the student and patient‐embedded actors.

**Table 3:** Simulation Scenarios

Roles for Embedded Actors	Scenario Overview and Background Information
**Scenario 1**	
Patient	A 26‐y‐old, gravida 2, para 1 term birth, 1 living child at 40 wk has a vaginal birth of a vigorous newborn and an immediate PPH due to uterine atony.
Student	A nervous beginning student lacks confidence and struggles to communicate.
**Scenario 2**	
Patient	A 28‐y‐old gravida 3, para 1 term birth, 1 abortion, 1 living child at 41 wk has a vaginal birth with a shoulder dystocia of a vigorous newborn and an immediate PPH due to uterine atony.
Student	An advanced student is confident and ready to take on additional responsibilities, but hesitates to move out of the way during the shoulder dystocia and is defensive during the debriefing discussion with the preceptor.

Abbreviation: PPH, postpartum hemorrhage.

## EVOLUTION OF THE PROGRAM

### Train the Simulation Facilitator Workshop for Preceptors

For this preceptor workshop to be sustainable, the Project aimed to create a cadre of preceptors who could facilitate the training and run the simulation for their colleagues at their clinical practices and facilities. The year following the initial workshop, the collaborating faculty created a *Simulation Facilitators Short Course for Midwifery Preceptors*. Participants in this course were preceptors who had already participated in the *Precepting Through Perinatal Emergencies Workshop* and had been practicing the methods taught in the course throughout the prior year. The objectives were to help the participants understand the components of high‐fidelity PRONTO‐Style Simulations, practice running and debriefing simulations, and prepare to cofacilitate simulations that emphasize preceptor‐learner communication. The group spent one day learning and practicing simulation basics and the next day training side‐by‐side with expert facilitators to train a new cohort of midwifery preceptors using the *Precepting Through Perinatal Emergencies* curriculum. This strategy proved successful, with 7 participants completing the training with the requisite skills, materials, and supplies. Each preceptor‐learner was given a simulation kit called a PRONTOPack, containing all the supplies and equipment needed to continue training their colleagues in their clinical settings.

### Preceptor Learning Platform

**Figure 2:**
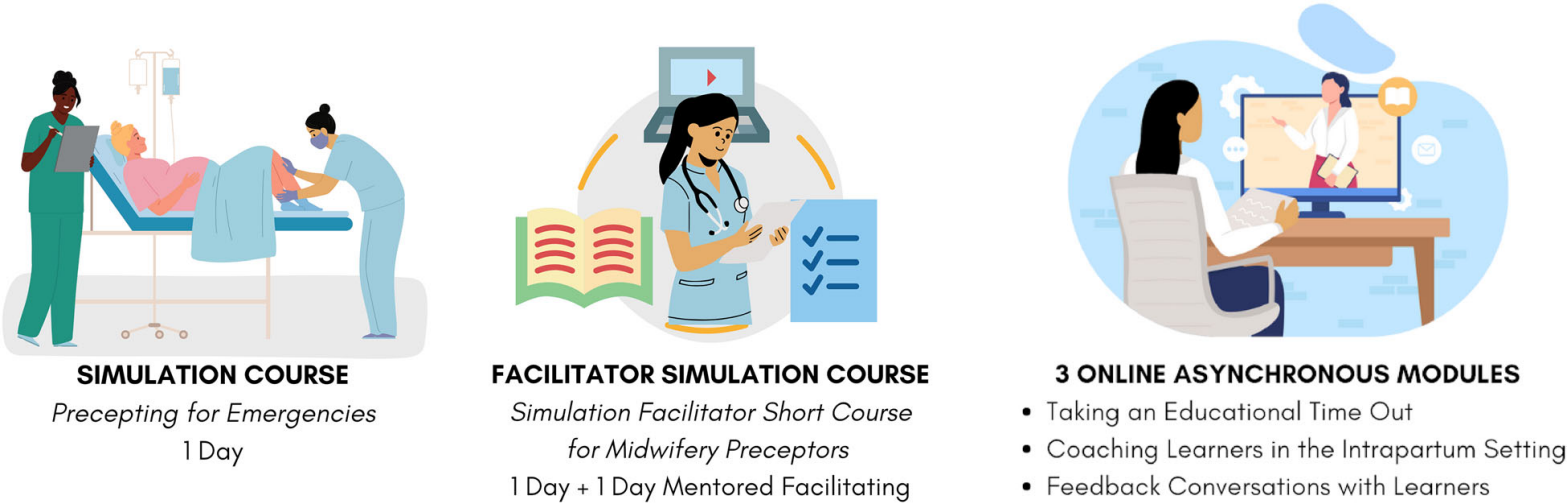
Evolution of the Program

Feedback from the New Jersey Midwifery Education Project's preceptor learning workshop participants demonstrated a desire for ongoing opportunities for preceptors to gain knowledge and skills around best practices in precepting, including during high‐stakes situations. The Project developed an online learning platform for preceptors housed in the Rutgers School of Nursing Center for Professional Development. The platform consists of a series of online modules, including 3 with embedded simulated demonstrations to introduce the concepts of the ETO, peer coaching, and debriefing to online participants. This asynchronous approach adds to midwives’ limited preceptor‐specific educational resources. See Figure 2 for an overview of the evolution of the program and a summary of the Educational Package.

## CONCLUSION

Successful growth of the midwifery workforce depends on cultivating a cadre of quality preceptors to educate and mentor midwifery students in the clinical setting. The *Precepting Through Perinatal Emergencies Workshop* is one example of an educational program using teaching and learning best practices specifically developed for midwifery preceptors. This training evolved to facilitate the sustainability of the specific skills taught, including a train‐the‐trainer model. Additionally, responding to participant feedback, the New Jersey Midwifery Education Project's preceptor workshop series has continued evolving to meet the educational needs of diverse New Jersey and national preceptors. Ideal next steps include an assessment of the impact of preceptor training on both preceptors and midwifery students, including modality (ie, in‐person or virtual) and session topics. Efforts to scale preceptor education, like the programming developed in New Jersey, including with funding and particularly for early career midwives, would facilitate the utilization of best practices for clinical education and training of the future midwifery workforce.

## CONFLICT OF INTEREST

The authors have no conflicts of interest to disclose.
